# A Malay Version of the International Society for the Prevention of Child Abuse and Neglect Screening Tool for Children: A Study of Its Validity and Reliability

**DOI:** 10.21315/mjms2020.27.4.9

**Published:** 2020-08-19

**Authors:** Mohd Faizul Sahaimi, Mohamad Najib Mat Pa, Fahisham Taib

**Affiliations:** 1 Paediatric Department, Hospital Universiti Sains Malaysia, Kubang Kerian, Kelantan, Malaysia; 2 Paediatric Department, School of Medical Sciences, Universiti Sains Malaysia, Kubang Kerian, Kelantan, Malaysia; 3 Department of Medical Education, School of Medical Sciences, Universiti Sains Malaysia, Kubang Kerian, Kelantan

**Keywords:** instrument, child abuse, validity, reliability, maltreatment

## Abstract

**Background:**

Childhood maltreatment is a global problem, for which the International Society for the Prevention of Child Abuse and Neglect (ISPCAN) has developed the Child Abuse Screening Tool–Child, Home Version (ICAST-CH) to obtain data concerning childhood maltreatment. The study aimed to translate the English version of the ICAST-CH into the Malay language and to assess its reliability and validity.

**Methods:**

The original English version of the ICAST-CH was first translated into the Malay language. Its content and face validity were tested among five independent individuals. A cross-sectional study using the Malay version (ICAST-CH-M) was then conducted with 255 students in a secondary school in Kota Bharu, Kelantan, Malaysia. The data collected was used to examine the instrument’s internal consistency and construct validity. The best ICAST-CH-M model was achieved after varimax rotation application.

**Results:**

The analysis showed that the Malay version of the ICAST-CH had satisfactory internal consistency, with Cronbach’s alpha ranging from 0.59–0.77. The exploratory factor analysis confirmed the validity of the underlying constructs into five domains in the Malay version, but they had to be re-classified as ‘physical and psychological abuse’, ‘neglect’, ‘sexual abuse’, ‘exposure to domestic violence’ and ‘exposure to community violence’.

**Conclusion:**

This study demonstrated that the ICAST-CH-M is satisfactorily reliable and valid for measuring child maltreatment in Malaysia.

## Introduction

Child maltreatment is an important medical problem experienced in the paediatric age group. It is a recognisable issue that exists worldwide, one involving extensive social problems (1–3). For example, a study that interviewed 2,869 young adults aged 18–24 years found that 16% of them had experienced maltreatment (3). In the East Asia and Pacific region, UNICEF reported that the overall prevalence rate of physical abuse ranged from 10%–30.3% (4). In 2017, the Malaysia Welfare Department documented 5,537 children who were identified for child protection (5). The incidence of such cases has been increasing annually, partly due to Malaysians’ increased awareness. As set forth in the Malaysian Child Act 2001, any public personnel and anyone who works with children are obligated to report any suspected case of child abuse to the managing authority for child protection investigation.

Many tools are available for the assessment and measurement of abuse and neglect of children, but none are ideal due to a lack of universality, cross-cultural understanding and standardisation of the instrument at a multinational level. These inconsistencies have influenced the actual prevalence, incidence and burden child abuse locally and internationally across societal milieus. One such instrument, the ICAST-CH, is used by the International Society for the Prevention of the Child Abuse and Neglect to measure child victimisation due to abuse and neglect (6). The instrument is a child self-report method with a moderate to high internal consistency score. A study in Taiwan that used a translated version produced almost similar to those for the original results in terms of reliability and validity (7). A few studies focusing on child abuse in Malaysia have been conducted, but none has captured the real prevalence of child maltreatment locally.

Translating and validating the ICAST-CH into the Malay language could thus be the first step to understanding child maltreatment and facilitating the building of a stronger foundation for a child-centred and child-friendly protection policy in Malaysia. The purpose of this study is to assess the validity and reliability of a translated Malay version of the ICAST-CH among Malaysian students (8–16).

## Methods

A cross-sectional validation study was carried out for two weeks in May 2016 at an urban secondary school (SMK Kota) located in the town of Kota Bharu, in the north-eastern part of Peninsular Malaysia.

### ICAST-CH Questionnaire

The ICAST-CH was designed by a group of international experts from different countries using a Delphi technique process and has been translated and tested in at least 20 languages. These multilanguage ICAST-CH questionnaires have been undergoing refinement based on feedback from translators and back-translators. The ICAST-CH has been found to identify high rates of child victimisation in all domains. Its rates of missing data are small and its internal consistency is moderate to high. Pilot testing demonstrated the feasibility of using child self-reporting as one strategy to assess child victimisation. Based on its development and validation process, the ICAST-CH is a very reliable tool for assessing child victimisation in a community.

The ICAST-CH initially consisted of 52 items and was designed for adolescents aged 11–18 years old. The instrument investigates the relationship between the child’s experience and his or her family environment. The questionnaire has high internal consistency for questions on exposure to violence (Q11–Q17), psychological abuse (Q18–Q25), neglect (Q26–Q31), physical abuse (Q32–Q40) and sexual abuse (Q41–Q46) with Cronbach’s alphas of 0.69, 0.78, 0.83, 0.77 and 0.72, respectively. Informed consent was obtained from the parent/caretakers and the students (subject). Informed consent was taken from the students prior to answering the questionnaire. Parental approval for each student was also obtained to ensure that the consent was valid as all the students were selected according to the predetermined inclusion criteria.

The self-administered ICAST-CH Malay (ICAST-CH-M) version questionnaire first underwent a forward translation into Malay and then a backward translation into English. The translating and pilot testing of the ICAST-CH-M was conducted in two phases.

#### Phase 1: The forward and backward translation of the questionnaire

**Figure 1:**
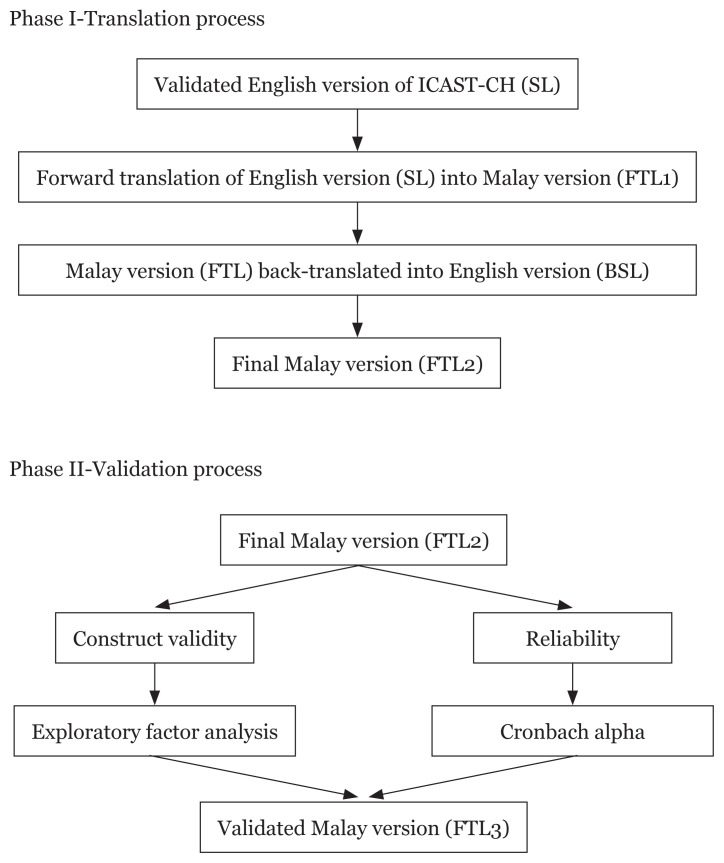
Flow chart of the study

The original ICAST-CH in English was translated into Malay based on forward translation guidelines. For the forward translation process, a bilingual professional linguist translator who was blinded to the study performed the translation from the English version (SL) to the Malay version (FTL). Following that, a backward translation from the FTL to the English version (BSL) was completed by a different professional bilingual translator who was also blinded and had no prior information regarding the original version of the questionnaire. The back-translated version (BSL) was then compared with the original SL to examine the translation equivalence (Figure 1). The translators used were proficient in both English and Malay.

The accuracy of the two translation versions was assessed by two independent bilingual speakers. Meaning and language equivalence were assessed with a 5-point Likert scale: 1 = extremely different, 2 = different, 3 = somewhat similar, 4 = similar and 5 = extremely similar. A score of at least 4 (representing similarity) was taken as appropriate equivalence. A group of experts (two paediatricians and two medical officers) that included the researchers reconciled the questions and composed the final Malay version (FTL2). Its face validity was tested on five independent individuals (house officers and medical students) who volunteered to participate in the pilot testing.

#### Phase 2: Testing ICAST-CH-M reliability and validity

The ICAST-CH-M consists of 51 items and has three sections; the first section consists of demographic data (9 items), the second section covers childhood victimisation (36 items) and the third section consists of open-ended questions (6 items). The childhood victimisation items include exposure to violence, psychological, physical, and sexual abuse, and neglect. Students were asked to specify the perpetrator and frequency of the abuse, when appropriate, for each of the 36 items on childhood victimisation.

Field testing of the ICAST-CH-M was conducted using a universal sampling method in a public school in Kota Bharu. The cross-sectional study was conducted in May 2016. We used a subject to item ratio of 5:1; the minimum sample required in this study was 175 as the original English version ICAST-CH has 35 questions. After considering a 10% drop out rate, the study’s final sample size was 193. The school is situated in a suburban area, easily accessible both by private and public transport. At least 1,000 students attend during the school term. Students 13–17 years of age were selected by a simple random sampling process; illiterate students were excluded. A random number was allocated for each class (Form 1–5). The number was written on a small piece of plain paper that was folded and mixed with the other numbers in a box. The researcher randomly selected numbers to determine the allocated classes for sampling. The process was repeated until the number of the subjects matched the determined sampling size. Data collection was then performed on four different occasions within a two-week period.

Instructions to the students were given in the local language (Malay language) by the principal researcher. Students were allowed to ask questions and clarify issues pertaining to the study prior to answering the questionnaires in their respective classroom environments. Students were given the option to opt out of the study at any time if they wished to do so. The principal investigator emphasised to the students that their information would be kept confidential. Data analysis was performed using SPSS (Statistical Package for Social Sciences) version 22 for OSX (SPSS Inc.). The demographic data consisted of the child’s age, gender and years of schooling, parental education, and household information. Cronbach’s alpha was used to assess the questionnaire’s reliability and an exploratory factor analysis was used to examine its construct validity.

For factor analysis, items on the questionnaire that did not exceed a 0.3 factor loading cut-off were deleted. Only factors with Eigenvalues greater than 1 were extracted and retained. A Kaiser-Meyer-Olkin (KMO) score of greater than 0.6 was used to ascertain sampling adequacy, and a Bartlett’s Test of Sphericity (BTS) of *α* < 0.05 was used for the correlation matrix.

## Results

### Sample Characteristics

A total of 255 students participated in the study, of whom 253 satisfactorily completed the questionnaires. Two students were excluded from the study due to not completing their questionnaires (less than 50% of the questions). The calculated sample size was 193, as described in the methodology. All of the students were Malay Muslims and girls comprised 51% (*n* = 129) of the cohort. The students’ ages ranged from 13–17 years with a mean age of 15.1 (SD 1.49) and the highest number of participants was from the 16-year-old group. A total of 212 (83.5%) students lived with both parents; 6 (2.4%) were identified as orphans at the time of the study (Table 1).

### Analysis of the Questionnaire

All of the students completed the questionnaire within 40 min. The response rate was excellent with 253 students successfully answering more than 80% of the questions (at least 40 completed items). Most of the unanswered questions were from three of the open-ended questions, item Q47 (Do you have any other experiences with being hurt that we have not already asked you about?), item Q48 (Do you have any suggestions for preventing violence against children?), and item Q51 (Is there anything else you would like to say about what happened to you or about filling in the questionnaire?). A majority of the questions in all subscales showed very low missing data, with only one exception, item Q20 on the psychological abuse subscale, for which the missing rate was slightly high at 2.8%. In general, the missing rate was 1.2%; four of the items had no missing data (Q12, Q13, Q25, and Q26) (Table 2).

Responses for each item on the subscales (exposure to violence, psychological, physical and sexual abuse, and neglect) were examined. The highest number of reported maltreatment were on items Q19 (*n* = 171) for psychological abuse, Q17 (*n* = 126) for exposure to violence and Q38 (*n* = 117) for physical abuse. Concerning the neglect items, most of the students reported experiences of feeling not cared for (Q29, *n* = 76), followed closely by not receiving adequate support or help (Q31, *n* = 74). For sexual abuse, all 6 items were reported positive, with the highest number on Q41 (*n* = 27). Four of the students indicated that they had made sex videos (Q45) and 6 of them reported they had experienced someone trying to have sex with them (Q46).

### Internal Consistency

Item Q36 (Has anyone burned or scalded you, including putting hot chilies or peppers in your mouth?) was removed because none of the students had any experience with such an event, making analysis of the item impossible. The internal consistency for each subscale after the items (Q 11, Q15, Q17, Q18, Q23, Q24, Q28, Q36 and Q38) were deleted ranged from 0.59–0.77 following EFA compression into 5-factor loading.

### Construct Validity

For construct validity, the KMO test showed a value of 0.739, exceeding the minimum value of 0.7 for an adequate sample. Based on the rotated component matrix, 9 items were eliminated because they failed to meet the minimum factor loading of 0.4 or above and did not contribute to a simple factor structure. A total of 25 items were retained. From the results of the exploratory factor analysis (EFA), five factor loadings were extracted. Cronbach’s alpha was recalculated based on the final constructs with the respective items. Factor 2 had the highest item loading with an internal consistency of 0.77.

Factor 1, labelled ‘physical and psychological abuse’, consisted of 8 items (Q19, Q20, Q25, Q32, Q33, Q34, Q38 and Q41) and had an internal consistency of 0.76, as well as good factor loadings ranging from 0.406–0.665. Factor 2, labelled ‘neglect’, included 7 items (Q14, Q21, Q22, Q29, Q30, Q31 and Q40) with factor loadings ranging from 0.443–0.784 and a Cronbach’s alpha of 0.77. Under factor 3, ‘sexual abuse’, 5 items (Q35, Q43, Q44, Q45 and Q46) were identified with factor loadings between 0.416–0.869 and a Cronbach’s alpha of 0.72. In factor 4, ‘exposure to domestic violence’, only 2 items (Q12 and Q13) were recognised; their factor loads were 0.622 and 0.734, and a Cronbach’s alpha of 0.61 was found. Factor 5 was labelled ‘exposure to community violence’ and had a Cronbach’s alpha of 0.59. This factor included 5 items (Q16, Q26, Q27, Q39 and Q42) with factor loadings between 0.486–0.631. Factor 4 and 5’s alphas were considered acceptable with nearly similar internal consistency (Table 3).

Table 4 illustrates the final number of deleted items in the ICAST-CH-M with satisfactory Cronbach’s alpha in each domain compared to the original English ICAST-CH (17).

## Discussion

This study is the first attempt to validate a cross-cultural, multidimensional tool specifically designed to examine children’s experiences of maltreatment using a population-based sample among adolescents specifically in Kota Bharu, Kelantan, Malaysia. The ICAST-CH has been used as a reliable tool in 20 countries.

The readability of the ICAST-CH-M items was appropriate for the students in the tested sample. Eighty-one percent of the students indicated that they understood the questions in the instrument and 94% of them indicated that the questions were easy to answer.

This was supported by low missing data in all but one item, as shown in Table 2. A pattern of large missing data could indicate that the questions were not readily understood or were ambiguous. Such concerns are particularly critical for a study that involves multinational, multilingual and multicultural respondents. Furthermore, any question that is regarded as threatening or sensitive can produce a high rate of missing data (6). To an Asian population, questions related to sexual behaviour are considered sensitive and offensive, but, interestingly, in our study the sexual abuse items had low missing data. This finding supported that the students were comfortable expressing themselves anonymously.

The ICAST-CH-M instrument is a self-report measurement tool aimed at exploring children’s past experiences. The initial reliability analysis of the ICAST-CH-M produced an overall score of 0.84, which was good, after excluding one item (Q36) with zero variance. However, in the subscale scores, the reliability scores were only acceptable, ranging from 0.59–0.77. The study was able to elicit loading for five factors; factors 1, 2 and 3 had Cronbach’s alpha of 0.76, 0.77 and 0.72, respectively. Factors 4 and 5 had satisfactory factor loadings with Cronbach’s alpha of 0.61 and 0.59, respectively. The satisfactory values were obtained after the removal of items with poor item-total correlation (< 0.3), which indicates satisfactory correlation with the overall scale. This was different from previous studies that possessed a good internal consistency with Cronbach’s alpha total scale of 0.72–0.90 (6, 7). In our study, the alpha coefficients for each item score were acceptable.

Reliability is a function of scores obtained for the particular administration of an instrument to a certain group of people. It is dependent on the sample and the scores obtained from that particular sample; alpha is a property of the scores on a test of a specific sample of tests (19). There are two main factors that affect coefficient alpha: the total test variance and the length of the test (number of items) (18). Other factors are poor interrelatedness between items, or heterogeneous constructs, and item difficulty (18, 19). Total test score variance is significantly affected by the homogeneity test of the sample. Increased heterogeneity of the group leads to increased variability in the total score, and thus increases the coefficient alpha, while the smaller the total test score variance, the smaller the coefficient alpha and vice versa.

Even though the students differed by gender and age, all the students were similar regarding attendance, ethnicity, religion and social background. This homogeneity might have contributed to the acceptable scores on coefficient alpha. Another factor was the difficulty of the test; more than 90% of the respondents indicated that the questions were not difficult. The degree of difficulty of the test, in general, affects the value of the alpha score.

The items’ construct validity was examined by exploratory factor analysis (EFA). The final analysis produced five constructs. The overall communality for items in each construct was acceptable (> 0.3), and factor loadings > 0.4 were found for all items in each construct following rotational analysis. A high factor loading indicated a high correlation between the item and construct. While there is no specific cut-off value for what reliability is acceptable, a minimum value of 0.60 is desirable for basic research evaluation. We consider two constructs (Factors 4 and 5) to have acceptable Cronbach’s alpha, 0.61 and 0.59, respectively. These two factors were labelled ‘exposure to violence’ items (Q12 and Q13) and ‘exposure to community violence’ items (Q16, Q26, Q27, Q39 and Q42).

Factor 1’s 8 items (Q19, Q20, Q25, Q29, Q30, Q32, Q34 and Q38) were a mixture of ‘physical and psychological abuse’. In factor 2, ‘neglect’, 5 items loaded satisfactorily (Q35, Q43, Q44, Q45 and Q46). Factor 3 included items (Q13, Q43, Q44, Q45 and Q 46) specifically from the ‘sexual abuse’ domain. After varimax rotation of the factor loading, items Q11, Q15, Q17, Q18, Q23, Q24, Q28, Q36 and Q37 were removed. Our study thus yielded 25 items which could be used in the Malaysian context. An EFA of the ICAST-CH-M produced five independent factors with satisfactory factor loadings and communality. The EFA was performed by fixing the number of extracted factors to five to match the original questionnaire, despite removing unfit items accordingly. The end analysis also found a satisfactory Cronbach’s alpha for each factor loading, despite factor 5 possessing a borderline alpha of 0.59. The low Cronbach’s alpha for factor 5 was perhaps influenced by the rigor of the translation and cross-adaptation during the content and face validation procedure.

The ICAST-CH was developed to enable international community child maltreatment assessment. Cross-cultural translation is required when an instrument is developed in another place and language and must be translated to be applied locally. It requires adjustments to fit the target culture rather than designing another instrument in the language of the culture. In the attempt to translate, the challenge is to acquire cross-cultural invariance for the instrument, i.e. the scale should be invariant regardless of linguistic and cultural differences. The added value for ICAST-CH-M is that following cross-cultural translation and examination of the tool, five item constructs have been identified suitable for the Malaysian environment. This should be followed up by a study to evaluate the new ICAST-CH-M.

The limitations of this study include the method of sampling (universal), a homogenous Malay population, similarity in demographic backgrounds and single institution (one public school) field testing. This can affect the generalisability of the study. A potential threat to the validity of the study is related to language translation. A translated instrument should undergo content, context, semantic and technical evaluation appropriate for a new setting and environment. Inconsistencies in the translation of English or Malay resulted in different understandings and interpretations of the intended questions. Selection bias may have also occurred in the expert panel selection.

## Conclusion

The overall results showed that the ICAST-CH-M has good construct validity and acceptable reliability. This is based on the EFA analysis finding each domain > 0.4 and overall Cronbach’s alpha in each subscale within the acceptable range. We conclude that the ICAST-CH-M is a valid tool for assessing childhood victimisation.

## Figures and Tables

**Table 1:** Demographic data of the students participated in the study

Data	Total (*n* = 253) *n* (%)
Gender	
Boy	124 (49.0)
Girl	129 (51.0)
Years of schooling (in years)	
7	35 (13.8)
8	31 (12.3)
9	45 (17.8)
10	57 (22.5)
11	74 (29.2)
12	11 (4.3)
Live with parents	
Father	4 (2.4)
Mother	31 (12.3)
Both	212 (83.8)
None of the parent	6 (2.4)

**Table 2:** Frequency and percentage to each item with missing data

Item no	Item	Response *n* (%)	Missing *n* (%)
	Exposure to violence (total)		
Q11	Adult used drugs then frightened	252 (99.6)	1 (0.4)
Q12	Adult shouted in a frightening way	253 (100)	0
Q13	Witnessed adult in home hit, kick, slap	253 (100)	0
Q14	Witnessed adult in home uses weapon	251 (99.2)	2 (0.8)
Q15	Someone close got killed near home	248 (98.0)	5 (2.0)
Q16	Seen people being shot, bombs, fighting or rioting	250 (98.8)	3 (1.2)
Q17	Something was stolen from home	250 (98.8)	3 (1.2)
	Psychological abuse (total)		
Q18	Scream	252 (99.6)	1 (0.4)
Q19	Insulted	252 (99.6)	1 (0.4)
Q20	Made you feel embarrassed	246 (97.2)	7 (2.8)
Q21	Wished you were dead	251 (99.2)	2 (0.8)
Q22	Threatened to abandon	252 (99.6)	1 (0.4)
Q23	Locked out of home	252 (99.6)	1 (0.4)
Q24	Threatened to hurt or kill you	252 (99.6)	1 (0.4)
Q25	Bullied by another child at home	253 (100)	0
	Neglect (total)		
Q26	Went hungry or thirsty	253 (100)	0
Q27	Inadequate clothes	251 (99.2)	2 (0.8)
Q28	Unmet medical need	249 (98.4)	4 (1.6)
Q29	Felt was not cared for	250 (98.8)	3 (1.2)
Q30	Felt unimportant	250 (98.8)	3 (1.2)
Q31	Inadequate support/help	250 (98.8)	3 (1.2)
	Physical abuse		
Q32	Pushed, grabbed, kicked	252 (99.6)	1 (0.4)
Q33	Hit, beat, spanked with hand	252 (99.6)	1 (0.4)
Q34	Hit, beat, spanked with object	252 (99.6)	1 (0.4)
Q35	Tried to choke, smother, or drown	251 (99.2)	2 (0.8)
Q36	Burned or scalded	251 (99.2)	2 (0.8)
Q37	Locked in small place	251 (99.2)	2 (0.8)
Q38	Pulled hair, pinched, twisted ear	252 (99.6)	1 (0.4)
Q39	Hold heavy load or exercise as punishment	250 (98.8)	3 (1.2)
Q40	Threatened with knife or gun	250 (98.8)	3 (1.2)
	Sexual abuse		
Q41	Talked to you in a sexual way	251 (99.2)	2 (0.8)
Q42	Showed pornography	252 (99.6)	1 (0.4)
Q43	Touched private parts	252 (99.6)	1 (0.4)
Q44	Made you look at their private parts or wanted to look at yours	250 (98.8)	3 (1.2)
Q45	Made a sex video of you	252 (99.6)	1 (0.4)
Q46	Tried to have sex with you	252 (99.6)	1 (0.4)

**Table 3:** The best factorial model of Malay ICAST-CH based on Exploratory Factor Analysis

Items		Rotated Factor Loadings				

		1	2	3	4	5
Q38:	*Menarik rambut anda, mencubit anda atau memulas telinga anda?* Pulled your hair, pinched you, or twisted your ear?	0.665				
Q32:	*Menolak, menyambar atau menendang anda?* Pushed, Grabbed, or Kicked you?	0.664				
						
Q19:	*Menggelar anda, bercakap perkara tidak baik atau mengutuk anda?* Called you names, said mean things or cursed you?	0.609				
Q25:	*Adakah anda dibuli (diejek, berasa malu) supaya anda berasa sedih atau tidak baik oleh kanak kanak lain di rumah?* Have you been bullied (teased, embarrassed) so that you feel sad or bad, by another child at home?	0.584				
Q33:	*Memukul, membelasah atau menampar anda menggunakan tangan?* Hit, beat, or spanked you with a hand?	0.580				
Q41:	*Membuatkan anda marah dengan bercakap kepada anda dengan cara seksual atau menulis perkara-perkara seksual tentang anda?* Made you upset by speaking to you in a sexual way or writing sexual things about you?	0.507				
Q20:	*Menyebabkan anda berasa segan/malu di hadapan orang lain dengan cara yang anda akan sentiasa berasa susah hati?* Made you feel ashamed/embarrassed in front of other people in a way you will always feel bad about?	0.437				
Q34:	*Memukul, membelasah atau menampar anda dengan tali pinggang, pemukul, kayu atau objek lain?* Hit, beat, or spanked you with a belt, paddle, a stick or other object?	0.406				
Q30:	*Berasakan bahawa anda tidak penting?* Felt that you were not important?		0.784			
Q29:	*Anda rasa tidak dipedulikan?* You did not feel cared for?		0.757			
Q21:	*Mereka mengatakan bahawa mereka harap anda mati/tidak pernah dilahirkan*? Said that they wished you were dead/had never been born?		0.723			
Q31:	*Berasakan bahawa tidak pernah ada sesiapa yang menjaga diri anda, menyokong anda, membantu anda apabila anda paling memerlukannya?* Felt that there was never anyone looking after you, supporting you, helping you when you most needed it?		0.591			
Q22:	*Mengancam untuk meninggalkan anda selama-lamanya atau membiarkan anda?* Threatened to leave you forever or abandon you?		0.515			
Q14:	*Pernahkah anda melihat sesiapa di dalam rumah anda menggunakan pisau, senjata api, kayu, batu atau benda lain untuk mencederakan atau menakutkan orang lain di dalam rumah?* Have you seen anyone in your home used knives, guns, stick, rocks or other things to hurt or scare someone else inside home?		0.452			
Q40:	*Mengancam anda dengan pisau atau senjata api* Threatened you with a knife or a gun		0.443			
Q46:	*Cuba untuk melakukan hubungan seks dengan anda apabila anda tidak mahu?* Tried to have sex with you when you did not want them to?			0.869		
Q45:	*Membuat video seks anda secara bersendirian atau dengan orang lain melakukan perkara seksual?* Made a sex video of you alone or with other people doing sexual things?			0.755		
Q43:	*Membuatkan anda melihat alat sulit mereka atau meminta melihat alat sulit anda?* Made you look at their private parts or wanted to look at yours?			0.729		
Q44:	*Menyentuh bahagian sulit anda atau membuat anda menyentuh alat sulit mereka?* Touched your private parts, or made you touch theirs?			0.659		
Q35:	*Mencekik anda, menekupkan muka anda dengan sesuatu atau melemaskan anda?* Choked you, smothered you or tried to drown you?			0.416		
Q13:	*Pernahkah anda melihat orang dewasa di dalam rumah anda memukul, menendang, menampar, menumbuk antara satu sama lain atau menyakiti antara satu sama lain secara fizikal dengan cara yang lain?* Have you seen adults in your home hit, kick, slap, punch each other or hurt each other physically in other ways?				0.734	
Q12:	*Pernahkah anda melihat orang dewasa di dalam rumah anda mencekik dan menjerit pada satu sama lain (bertengkar) dengan cara yang menakutkan anda?* Have you seen adults in your home shouting and yelling at each other (arguing) in a way that frightened you?				0.622	
Q26:	*Adakah anda rasa anda tidak mendapat makanan yang mencukupi (kelaparan) dan/atau minum (kehausan) walaupun ia cukup untuk semua orang?* Do you feel that you did not get enough to eat (went hungry) and/or drink (were thirsty) even though there was enough for everyone?					0.631
Q16:	*Adakah anda tinggal di tempat di mana anda telah melihat orang ditembak, bom meletup, orang berjuang, atau rusuhan?* Have you lived somewhere where you have seen people being shot, bombs going off, people fighting, or rioting?					0.630
Q27:	*Memakai pakaian koyak, kotor atau kain yang tidak cukup panas/ terlalu panas, kasut yang terlalu kecil walaupun terdapat cara untuk mendapatkan yang lebih baik/yang baru?* Have to wear dirty, torn clothes, or clothes that were not warm enough/too warm, shoes that were too small even though there were ways of getting better/new ones?					0.628
Q39:	*Menetapkan anda berada pada satu kedudukan memegang beban berat atau beban lain atau mengarah anda melakukan senaman sebagai hukuman?* Making you stay in one position holding a heavy load or another burden or making you do exercise as punishment?					0.491
Q42:	*Membuatkan anda menonton video seks atau melihat gambar gambar seks dalam majalah atau komputer apabila anda tidak mahu?* Made you watch a sex video or look at sexual pictures in a magazine or computer when you did not want to?					0.486
Eigenvalues		3.228	3.136	2.854	2.015	1.933
% of variance		11.954	11.613	10.569	7.464	7.160

**Table 4:** Results of overall internal consistencies for ICAST-CH-M

Construct	Original items no in ICAST-CH (English)	Cronbach’s-α (before items deleted) #	New item no in ICAST-CH (Malay)	Cronbach’s-α (after items deleted)	Final construct in Malay ICAST- CH subscale*
Physical abuse	9	0.77	8	0.76	I
Neglect	6	0.83	7	0.77	II
Sexual abuse	6	0.72	5	0.72	III
Psychological abuse	7	0.78	2	0.61	IV
Violence exposure	7	0.69	3	0.59	V
Total scale	35		25		

Notes:

* I = physical and psychological abuse; II = neglect; III = sexual abuse; IV = exposure to domestic violence; V = exposure to community violence;

# (17)
